# Finite Element Study of a Lumbar Intervertebral Disc Nucleus Replacement Device

**DOI:** 10.3389/fbioe.2016.00093

**Published:** 2016-12-01

**Authors:** Jessica S. Coogan, W. Loren Francis, Travis D. Eliason, Todd L. Bredbenner, Brian D. Stemper, Narayan Yoganandan, Frank A. Pintar, Daniel P. Nicolella

**Affiliations:** ^1^Southwest Research Institute, San Antonio, TX, USA; ^2^Spinal Stabilization Technologies, San Antonio, TX, USA; ^3^Department of Neurosurgery, Medical College of Wisconsin, Milwaukee, WI, USA; ^4^Clement J. Zablocki Veterans Affairs Medical Center, Milwaukee, WI, USA

**Keywords:** finite element modeling, finite element analysis, intervertebral disc, degenerative disc, nucleus replacement, spine biomechanics

## Abstract

Nucleus replacement technologies are a minimally invasive alternative to spinal fusion and total disc replacement that have the potential to reduce pain and restore motion for patients with degenerative disc disease. Finite element modeling can be used to determine the biomechanics associated with nucleus replacement technologies. The current study focuses on a new nucleus replacement device designed as a conforming silicone implant with an internal void. A validated finite element model of the human lumbar L3–L4 motion segment was developed and used to investigate the influence of the nucleus replacement device on spine biomechanics. In addition, the effect of device design changes on biomechanics was determined. A 3D, L3–L4 finite element model was constructed from medical imaging data. Models were created with the normal intact nucleus, the nucleus replacement device, and a solid silicone implant. Probabilistic analysis was performed on the normal model to provide quantitative validation metrics. Sensitivity analysis was performed on the silicone Shore A durometer of the device. Models were loaded under axial compression followed by flexion/extension, lateral bending, or axial rotation. Compressive displacement, endplate stresses, reaction moment, and annulus stresses were determined and compared between the different models. The novel nucleus replacement device resulted in similar compressive displacement, endplate stress, and annulus stress and slightly higher reaction moment compared with the normal nucleus. The solid implant resulted in decreased displacement, increased endplate stress, decreased annulus stress, and decreased reaction moment compared with the novel device. With increasing silicone durometer, compressive displacement decreased, endplate stress increased, reaction moment increased, and annulus stress decreased. Finite element analysis was used to show that the novel nucleus replacement device results in similar biomechanics compared with the normal intact nucleus.

## Introduction

Surgical treatments for symptomatic degenerative disc disease (DDD) refractory to conservative measures include spinal fusion, discectomy, and total disc replacement, with spinal fusion being the current standard of care. However, fusion fundamentally alters the biomechanics of the spine, often resulting in reduced range of motion that can lead to degenerative effects in the motion segments adjacent to the fusion (Harris and Wiley, 1963; Lee, 1988; Aota et al., 1995). Discectomy and total disc replacement are alternatives to spinal fusion, but higher quality prospective, controlled, long-term follow-up studies are necessary to show that alternative treatments have a clinically relevant difference compared with fusion (Loupasis et al., 1999; van den Eerenbeemt et al., 2010; Jacobs et al., 2012). Nuclear replacement devices have been developed as a more minimally invasive treatment that could potentially correct the effects of DDD without negatively altering the biomechanics of the vertebral segment. However, previous nucleus replacement technologies have resulted in adverse outcomes such as extrusion through the annulus fibrosus and subsidence into the vertebral bodies (Bertagnoli and Schonmayr, 2002; Klara and Ray, 2002; Allen et al., 2004; Bono and Garfin, 2004). New nucleus replacement devices must be evaluated for the ability to restore the natural biomechanics of the spine without adverse outcomes.

Finite element analysis can be used to determine if a nucleus replacement device implanted in an enucleated intervertebral disc (IVD) is capable of restoring the biomechanical properties of a lumbar motion segment. Several previous finite element studies have investigated the effect of nucleus replacement devices. Earlier finite element models evaluated the effect of axial compression on simplified models of the IVD. The studies compared the biomechanics associated with the normal IVD, IVD with nucleotomy, and IVD with linearly elastic nucleus replacements of various sizes and material properties (Meakin et al., 2001; Joshi et al., 2009; Strange et al., 2010). Nucleotomy was shown to change the stress distribution within the annulus and lead to inward bulging of the annulus under axial compression, where the natural IVD bulges outward. A nucleus implant that completely filled the nuclear chamber restored the axial compressive mechanical properties, normal annulus stress distribution, and annulus bulge (Meakin et al., 2001; Strange et al., 2010). Variations in the geometry of the implant were found to contribute more to the biomechanics than changes in properties (Joshi et al., 2009). More recent studies utilized more sophisticated models where the geometry was based on medical imaging data, and the models were loaded under compression and rotation (Rundell et al., 2009; Dahl et al., 2010; Schmidt et al., 2014). These studies showed that a fully conforming implant more accurately replicates the native biomechanics and that an overly stiff device can lead to adverse outcomes, such as subsidence (Rundell et al., 2009; Dahl et al., 2010; Schmidt et al., 2014).

A new nucleus replacement device has been designed as a conforming silicone implant with a central cavity. The central cavity of the nucleus replacement device provides three functions. First, the central cavity is filled with contrast during the procedure, which allows for visualization and positioning of the device. Second, the central cavity acts as a pressure transducer during the filling of the outer cavity with silicone. When the nucleus cavity has been completely filled the silicone transmits pressure to the contrast filled central cavity, which is then registered on a pressure gauge. This allows the surgeon to know that the fill has been completed. Finally, once the silicone has cured and the contrast removed a void is left in the central cavity. When the disc is loaded, this void allows the silicone to displace inward, compressing the central cavity. With a solid implant, all displacement of the implant under loading is outward against the annulus. By allowing for some inward displacement into the central cavity, the risk of extrusion into the annular access may be reduced. Additionally, the central cavity may reduce the amount of stress at the center of the endplate, which is most vulnerable to subsidence and fracture (Bono and Garfin, 2004).

The aim of this study was to perform a finite element analysis of an L3–L4 motion segment to investigate the biomechanical response of a novel IVD nucleus replacement device with a central cavity and to predict the effects of the device on the kinematics, endplate stresses, and annulus stresses when compared to a normal IVD and solid silicone implant. Material testing of silicone samples was utilized to fit a non-linear material model, so that the device mechanics were more accurately represented. The finite element model was also used to perform a sensitivity analysis of the material properties of the device in order to determine the resulting biomechanics associated with different device material selection.

## Materials and Methods

A three-dimensional finite element model of the L3–L4 lumbar spine segment was developed from medical imaging data. Additional models were constructed with various nucleus replacement devices in place of the normal nucleus. The model was loaded with axial compression followed by flexion, extension, lateral bending, or axial rotation. A sensitivity analysis was performed on the design parameters of the nucleus replacement device. The finite element code LS-Dyna^®^ (LSTC, Livermore, CA, USA) was used throughout the study.

### Generation of Normal Baseline Model

A three-dimensional finite element model of the L3–L4 lumbar spine segment was developed from CT images of five postmortem human lumbar spines (mean age of 42.2 ± 13.7 years) (Stemper et al., 2011). An average model was created from the five sets of image data using statistical shape modeling (SSM) methods that are described in detail elsewhere (Bredbenner et al., 2010, 2014; Nicolella and Bredbenner, 2012). SSM provides a parametric framework for representing variability in a large number of individual complex anatomical shape instances within a population (Lorenz and Krahnstover, 2000). Briefly, the vertebrae for each of the five individuals were semiautomatically segmented (Seg3D, The Center for Integrative Biomedical Computing, University of Utah, Salt Lake City, UT, USA) and meshed using four node tetrahedral elements (MATLAB R2012a, The Mathworks Inc., Natick, MA, USA). The first individual was arbitrarily chosen as the template mesh, and this template mesh was registered to the vertebral geometry of each of the other individuals using a coherence point drift algorithm (Myronenko and Song, 2010). Through using the coherence point drift algorithm, the resulting vertebral surface geometry for each individual was then defined by the same mesh definition, and the vertices of the mesh were positioned at corresponding anatomic locations between individuals. An average model was defined by averaging vertex positions across the individuals. The properties of the vertebral bodies were modeled with an isotropic elastic perfectly plastic material model (LS-Dyna, LSTC, Livermore, CA, USA) based on the average QCT gray scale values across the individuals. The gray scale values were binned into 20 levels, and the corresponding elastic modulus and yield strength were based on empirical relationships (Kopperdahl et al., 2002). The cortical bone and cartilaginous endplate were modeled using shell elements with a thickness of 0.35 mm. An elastic material model was used to model the cartilaginous endplate and the cortical bone, with properties given in Table 1.

**Table 1:** **Material properties of the finite element model**.

Structure	Property	Value
Cartilaginous endplate	Young’s modulus (Schmidt et al., 2007)	24 MPa
Cortical bone	Young’s modulus (Rundell et al., 2009; Dahl et al., 2010)	12 GPa
Nucleus pulposus	Bulk modulus (Meakin, 2001)	1.7 GPa
Annulus	Young’s modulus (Shirazi-Adl et al., 1986)	4.6 MPa
	Poisson’s ratio (Shirazi-Adl et al., 1986)	0.45

**Figure 1:**
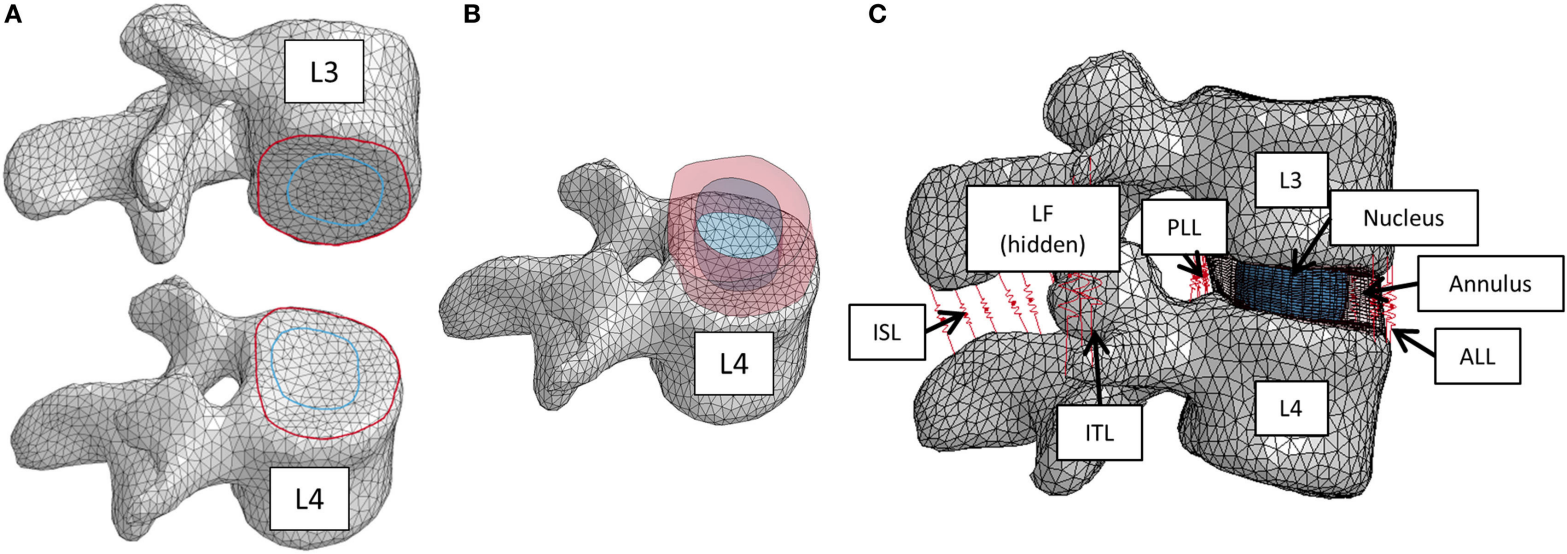
**(A)** Splines on the inferior L3 vertebrae and superior L4 vertebrae that were used to create the intervertebral disc. **(B)** Lofted splines created the geometry of annulus (red) and nucleus (blue). **(C)** Finite element model of the L3–L4 vertebral motion segment. Ligaments are shown as red springs, with abbreviations ALL, anterior longitudinal ligament; PLL, posterior longitudinal ligament; ISL, interspinous ligament; ITL, intertranverse ligament; LF, ligamenta flava (hidden from view).

To create the IVD, splines were generated based on the segmented image on the inferior L3 vertebral body endplate and the superior L4 vertebral body endplate that represented the annulus and nucleus boundaries on the adjacent endplates. The adjacent splines were lofted together to create the outer annulus boundary and the interface between annulus and nucleus (Figures 1A,B). The resulting geometry was meshed with eight node hexagonal elements (TrueGrid, XYZ Scientific Applications, Inc., Livermore, CA, USA). Connection between the disc and the endplate was made using a tied node to surface algorithm (LS-Dyna, LSTC, Livermore, CA, USA). The disc annulus was modeled using an isotropic material model, and the disc nucleus was modeled as a linear fluid (LS-Dyna, LSTC, Livermore, CA, USA), with properties given in Table 1. Facet joint cartilage was modeled by projecting the facet subchondral bone surface of the adjacent vertebrae outward along the surface normals to form a single layer of cartilage elements on each opposing facet surface. The cartilage thickness on each opposing facet was iteratively determined to maximize joint contact without facet cartilage surface interference. A penalty-based contact algorithm was used to model frictionless contact between facet surfaces (LS-Dyna, LSTC, Livermore, CA, USA). Ligaments (anterior longitudinal ligaments, posterior longitudinal ligaments, interspinous ligaments, ligamentum flavum, intertransverse ligaments, and joint capsule) were modeled using non-linear, tension-only, discrete spring elements to connect selected nodes on adjacent vertebrae (LS-Dyna, LSTC, Livermore, CA, USA). Material properties for the ligaments were derived from the ligament stiffness data from Pintar et al. (1992). The complete finite element model is shown in Figure 1C.

### Generation of Implant Models

**Figure 2:**
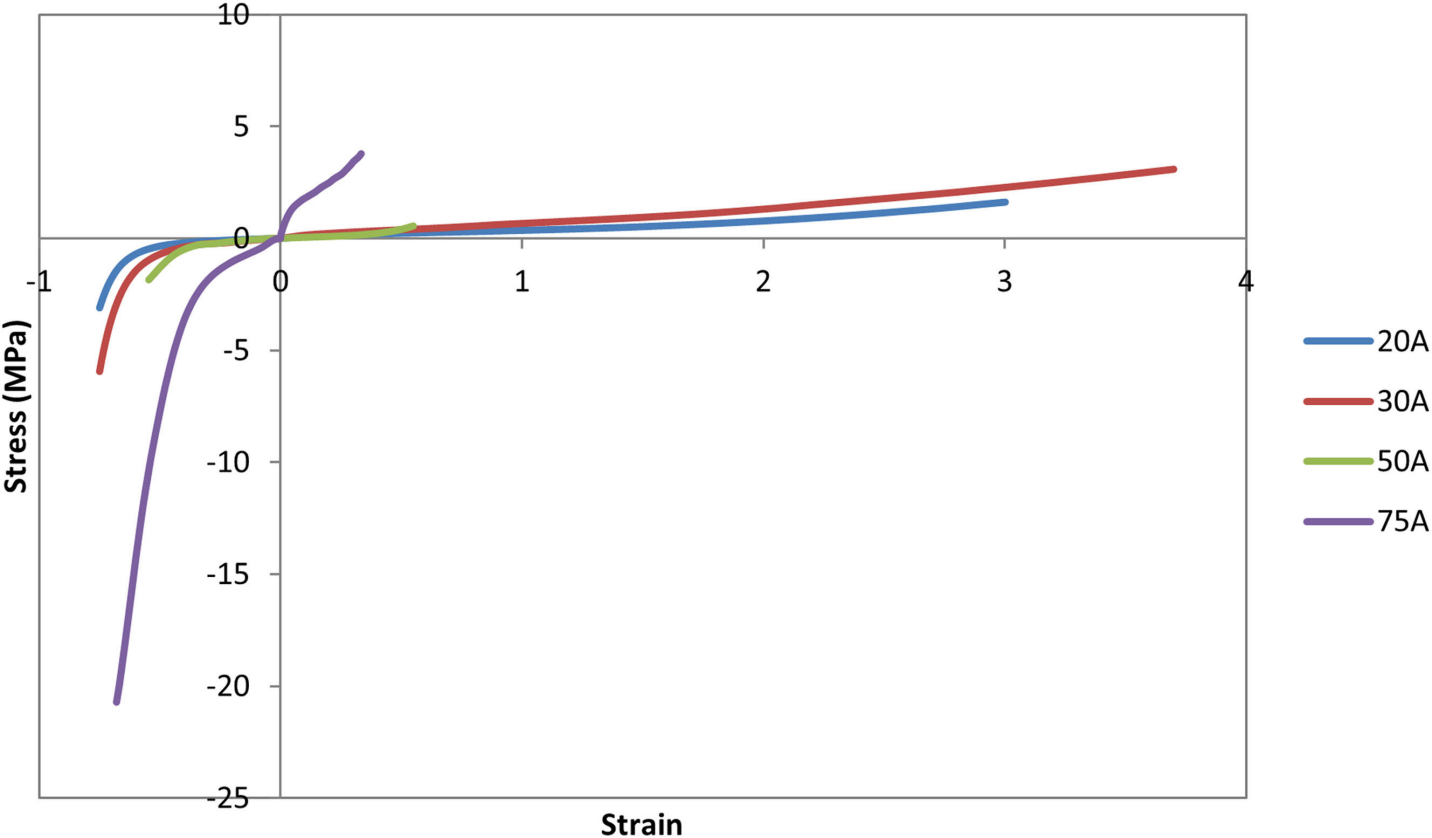
**Load curves used for the simplified rubber material model for each silicone durometer**. Load curves were determined by fitting the material behavior to experimental force–displacement data of silicone samples of the specified durometer.

**Figure 3:**
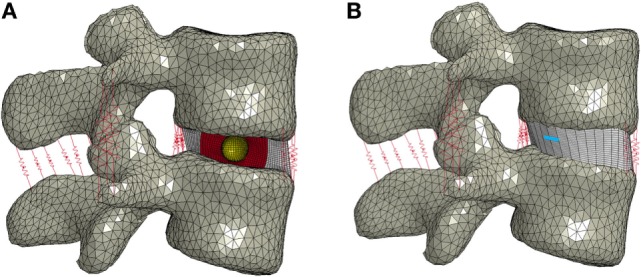
**(A)** Finite element model of the L3–L4 motion segment with the nucleus replacement device in place of the normal nucleus. A cross section of the disc annulus and device is shown, with the outer chamber of the device shown in red and the void inner chamber shown in yellow. **(B)** Finite element model showing the location of the annulus slit in blue.

The nucleus replacement implant is comprised of an outer chamber that is filled with silicone that cures *in situ* and becomes solid and an empty inner chamber that allows for inward deflection when the device is loaded. Using the baseline model, a new mesh representing the implant was used in place of the normal nucleus. An empty ellipsoid with a volume of 0.4 cc, major axis diameter of 11 mm, and minor axis diameter of 8 mm was introduced in the center of the IVD to represent the inner chamber of the device. The shape and volume of the ellipsoid were design decisions that were hypothesized to best replicate the biomechanics of the normal spine. The void was modeled with a simple pressure–volume relationship, with the volume contained by the empty ellipsoid decreasing linearly with increasing pressure (LS-Dyna, LSTC, Livermore, CA, USA). The area between the vertebrae, annulus, and inner chamber was space filled with hexahedral elements to represent the outer chamber of the device (TrueGrid, XYZ Scientific Applications, Inc., Livermore, CA, USA). A simplified rubber material model defined by a single load curve (LS-Dyna, LSTC, Livermore, CA, USA) was utilized for the outer chamber, where the load curve was determined by fitting the material behavior to experimental force vs. displacement data up to the point of failure (Figure 2). Experimental data were obtained from uniaxial compression and tensile testing of silicone rubber samples of varying durometers (data not shown). For the testing of Shore 20A silicone, 10 compression tests and 10 tension tests were performed with average coefficient of variation (CV) across the loading history of 10%, while 5 compression and 5 tension tests were performed on Shore 30A silicone with average CV of 16%. For Shore 50A and 75A, two compression and two tension tests were performed for each durometer, with average CV of 14 and 7%, respectively. Based on physician feedback, the initial configuration of the device utilizes Shore 20A durometer silicone in the outer chamber, which was represented in the computational model with the material model representing the Shore 20A load curve. Shell elements were defined around the entire surface of the inner chamber and the outer chamber to represent the silicone membranes of the device. The material model representing the Shore 30A load curve was used for the shell elements. An additional model was also constructed to simulate the resulting damage to the annulus after insertion of the device. The delivery of the device will be performed through progressive dilation and stretching of the annulus fibers with the intention that the fibers will return to their original orientation and reduce the size of the incision. The final outcome of this insertion was modeled by introducing a slit in the right posterior quadrant of the annulus of the model incorporating the device. Computationally, a 5.5-mm length slit was simulated by removing the connection of a set of two elements through the thickness of the annulus. The finite element model that incorporates the nucleus replacement device is shown in Figure 3A, and the location of the annulus slit is shown in Figure 3B. A model representing a solid implant was constructed by removing the inner void of the device, so that the entire nucleus was represented using the Shore 20A silicone material model described previously. In total, seven different models were created. Six of the models incorporated different disc nucleus representations, which were the normal nucleus, Shore 20A, 30A, 50A, 75A implants with inner chamber void, and Shore 20A solid implant. The seventh model utilized the Shore 20A implant with inner chamber void and had the annulus slit that represented the outcome after implantation.

### Simulation

Loading conditions were based on the ASTM F2423 standard for testing of lumbar IVD prostheses (ASTM Standard F2423-11, 2011), where an axial compressive load is applied followed by a specified rotation (Table 2). All simulations were performed using LS-Dyna^®^ (LSTC, Livermore, CA, USA).

**Table 2:** **Model boundary conditions for the axial load and prescribed displacement taken from ASTM F2423**.

	Axial load (*N*)	Rotation (°)
Flexion/extension	1200	±7.5
Axial rotation	1200	±3
Lateral bending	1200	±6

For all models, the L4 vertebra was constrained in all directions. Sliding contact was defined between the facets. The inferior and superior boundaries of the annulus and nucleus or device were constrained to the endplates. For the baseline normal case, the annulus and nucleus shared nodes at the interface, but for the implant models, sliding contact was defined between the annulus, textile band, and implant. A mesh convergence study was performed using the ASTM loading conditions to result in <10% change in the maximum mid-plane annulus stress, resulting in a baseline model with 31,185 nodes and 80,800 elements, with an average element characteristic length of 1.5 mm for the bone and 0.5 mm for the disc.

### Model Validation

The baseline model was quantitatively validated against experimental range of motion data from pure moment loading in axial rotation, lateral bending, and flexion and extension using methods from Barnes et al. (2009). Four male and two female lumbrosacral spines (T12–S1) were obtained from unembalmed postmortem human subjects with a mean age of 26.3 years (21–38 years) with approval by the Research and Development Committee at the Zablocki VA Institution. The T12 and S1 vertebral bodies were potted in polymethylmethacrylate (PMMA) such that the L3–L4 IVD was parallel to both fixtures. Pure moments were applied using a system of masses and pulleys, and forces were recorded with a six-axis load cell (Robert A Denton Inc., Rochester Hills, MI, USA) mounted between the lower PMMA fixation and the test frame. Pure flexion, extension, left and right lateral bending, and left and right axial rotation were statically applied in steps up to 6.0 Nm. The load cell was used to confirm purity of the applied moment. Stereophotogrammetry was implemented to determine specimen kinematics *via* a nine camera motion analysis system (Vicon Corp., Oxford Metrica, Oxford, England). Displacements of three non-collinear markers placed on pins inserted into each vertebral body were tracked. A motion analysis model was created to compute three-dimensional Euler rotations of each vertebra relative to the inferior vertebra.

In order to account for the variability in the lumbar soft tissue material property data, a probabilistic analysis was performed using the mean and SD data for the ligament and disc material properties. A scaling factor was introduced for the ligament force–displacement curves, where the mean was 1 and the variability was based on the experimental variability in ligament stiffness (Pintar et al., 1992). A lognormal distribution was used for the scaling factor to avoid negative scaling factors. Lacking experimental data, a 10% CV was assumed for the disc annulus Young’s modulus, where the modulus was also defined using a lognormal distribution to avoid negative values. The probabilistic analysis software NESSUS^®^ (NESSUS^®^, v. 9.7, Southwest Research Institute, San Antonio, TX, USA) was used to perform the probabilistic analysis. For the probabilistic analysis, 100 Latin hypercube samples (LHS) were used to determine the probabilistic response for each of the pure moment motion simulations.

The use of a quantitative metric provides a measure of agreement that is not accounted for using the standard graphical method of plotting the simulated response with corridors derived from experimental data. The probabilistic error metric is determined by defining the model error Z as the absolute error between the model prediction and experimental response (Thacker and Paez, 2014).

Z=Ymod−YexpE[Yexp],

where *Y*^mod^ and *Y*^exp^ are Gaussian random variables for the model and experiment, respectively. The model error Z is also a random variable, where the CDF of |Z| is given as

p=P(|Z|≤z).

The error metric can be reported either at a specified probability level or at a specified error level. At a specified probability level, for example, 90%, a statement can be made that there is a 90% probability that the error will not exceed the calculated error metric. Alternatively, the error metric value can be specified, for example, 10% error, and the probability that the error metric value would not exceed 10% can be calculated. To calculate the probabilistic error metric, an additional analysis was performed in NESSUS where the experimental response was also included as a random variable in the LHS analysis. Then, for each LHS, the value Z was directly calculated. NESSUS then generated the distribution of Z, allowing the probabilistic error metric to be calculated.

In order to provide a benchmark error value, the error at 90% probability was calculated assuming the model matched the experimental mean and variability perfectly. When the experimental results have a high level of variability, the probabilistic error metric will also be high even with a perfect match. Then, for the finite element model, the probability, *p*, that the benchmark error (*e*_benchmark_) was not exceeded was calculated using the equation:

p=P(|Z|≤ebenchmark).

### Sensitivity Analysis

A sensitivity analysis was performed to investigate the effect of a key design parameter of the device, the silicone durometer. The initial properties of the device utilized the material model representing silicone durometer of Shore 20A to fill the outer chamber, and the sensitivity analysis modified the material model to represent durometers of Shore 30A, 50A, and 75A while holding all other variables constant.

### Data Analysis

Model characterization was performed for both the axial compression loading step and the complete loading (compression followed by rotation). For axial compression alone, disc height loss (displacement of the L3 vertebra) and endplate stress (contact pressure between the disc and L3 endplate) were determined. For the complete loading, the reaction moment required to achieve the desired rotation and the stress in the annulus were computed. Specifically, the maximum transverse mid-plane stress on the interior surface of the annulus was determined. Mid-plane stress was chosen to minimize the effect of contact at the vertebrae–annulus interface. The interior surface of the annulus was chosen since that portion of the annulus is in contact with the nucleus or implant.

## Results

**Figure 4:**
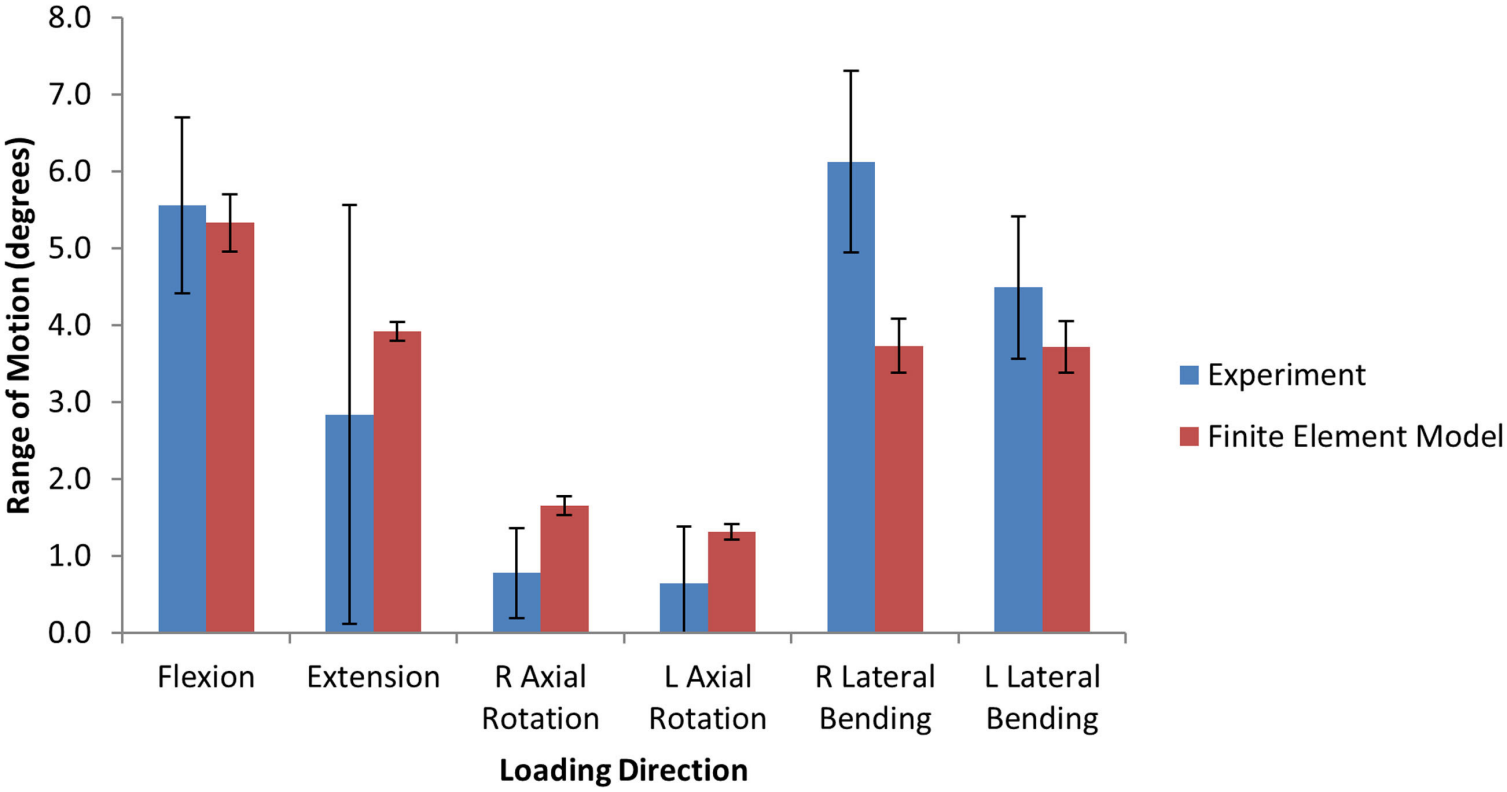
**Bar graphs providing a qualitative comparison the range of motion between the finite element model and the experimental data under 6.0 Nm pure moment loading**.

The experimental range of motion data used for model validation is given in Table 3. Figure 4 shows the qualitative comparison of means of the experimental and simulated responses, where the mean simulated response falls within the experimental corridors for all motions except right axial rotation and right lateral bending. For the quantitative validation metric, benchmark errors were very large, indicating high variability in the experimental data (Table 4). For the simulation, probability that the benchmark errors was not exceeded was highest for flexion and extension and lowest for right and left lateral bending, with higher probabilities indicating a greater degree of agreement between the experimental and simulated responses (Table 4).

**Table 3:** **Experimental range of motion data (degrees) for pure moment loading of the lumbar spine**.

Load direction	T12–L1	L1–L2	L2–L3	L3–L4	L4–L5	L5–S1
Flexion	4.5 (1.99)	3.22 (1.69)	4.68 (2.45)	5.56 (1.15)	6.47 (1.23)	8.98 (2.39)
Extension	1.6 (1.33)	1.67 (0.97)	2.02 (0.83)	2.84 (2.72)	2.97 (1.76)	2.75 (2.32)
R axial rotation	1.19 (0.7)	0.77 (0.44)	0.67 (0.5)	0.78 (0.58)	1.37 (0.53)	1.72 (1.28)
L axial rotation	1.36 (0.23)	0.62 (0.19)	0.78 (0.51)	0.65 (0.73)	1.54 (1.54)	1.67 (1.58)
R lateral bending	4.01 (1.35)	4.95 (0.7)	4.46 (1.12)	6.12 (1.18)	6.08 (1.71)	4.14 (2.42)
L lateral bending	3.79 (0.73)	3.96 (0.75)	4.41 (0.97)	4.49 (0.93)	5.44 (2.25)	4.41 (1.21)

*Data given as mean (SD)*.

**Table 4:** **Quantitative benchmark error values and the probability (*p*) of the finite element result error being less than the benchmark value**.

Motion	*e*_benchmark_ (%)	FEM *p* = *P*(|Z| ≤ *e*_benchmark_) (%)
Flexion	48	98
Extension	222	97
R axial rotation	172	81
L axial rotation	260	91
R lateral bending	45	61
L lateral bending	48	77

**Figure 5:**
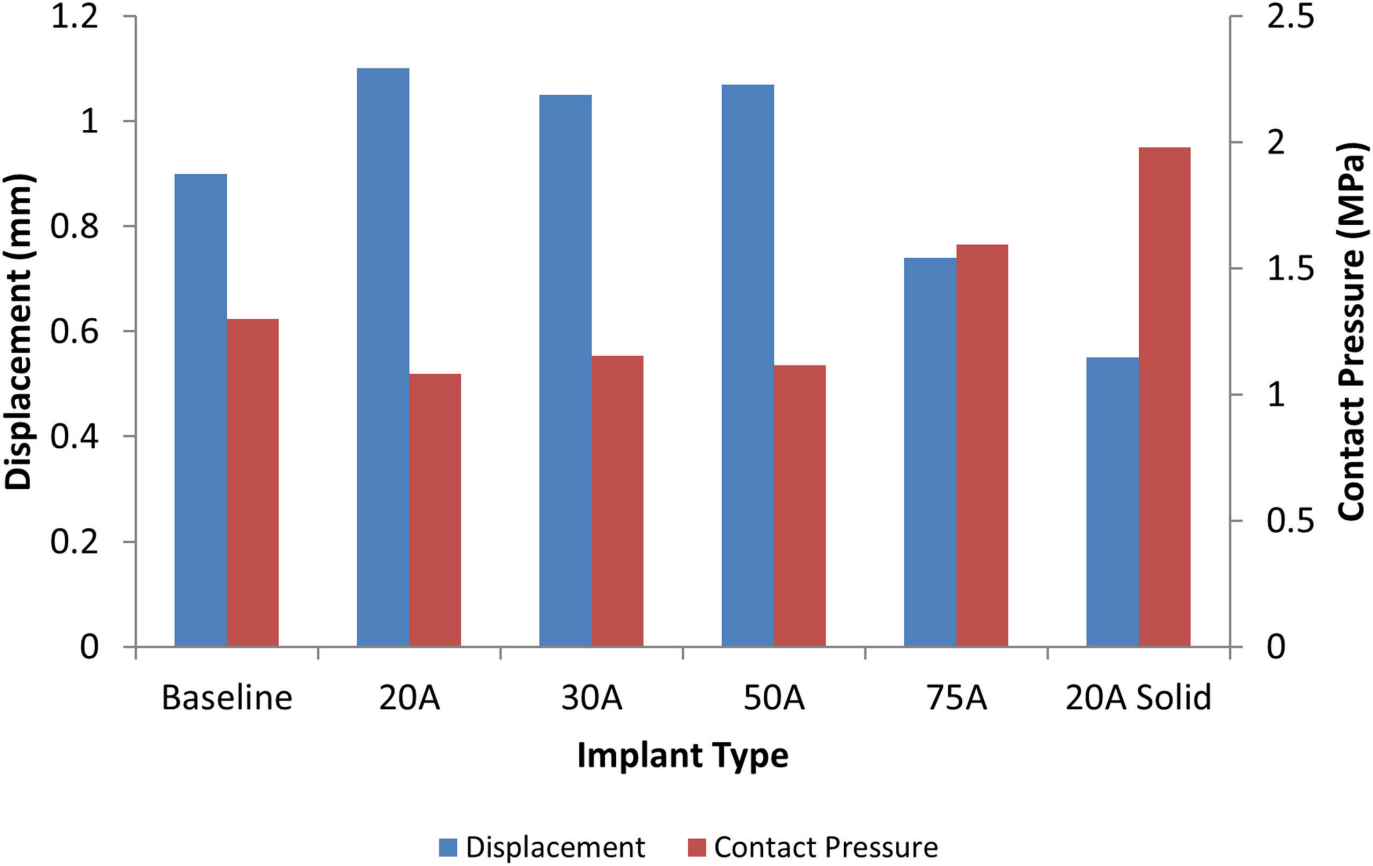
**Comparison of L3 vertebrae displacement (blue) and contact pressure (red) with a 1200 N axial load with varying silicone durometers for the implant with chamber and the solid Shore 20A implant**.

**Figure 6:**
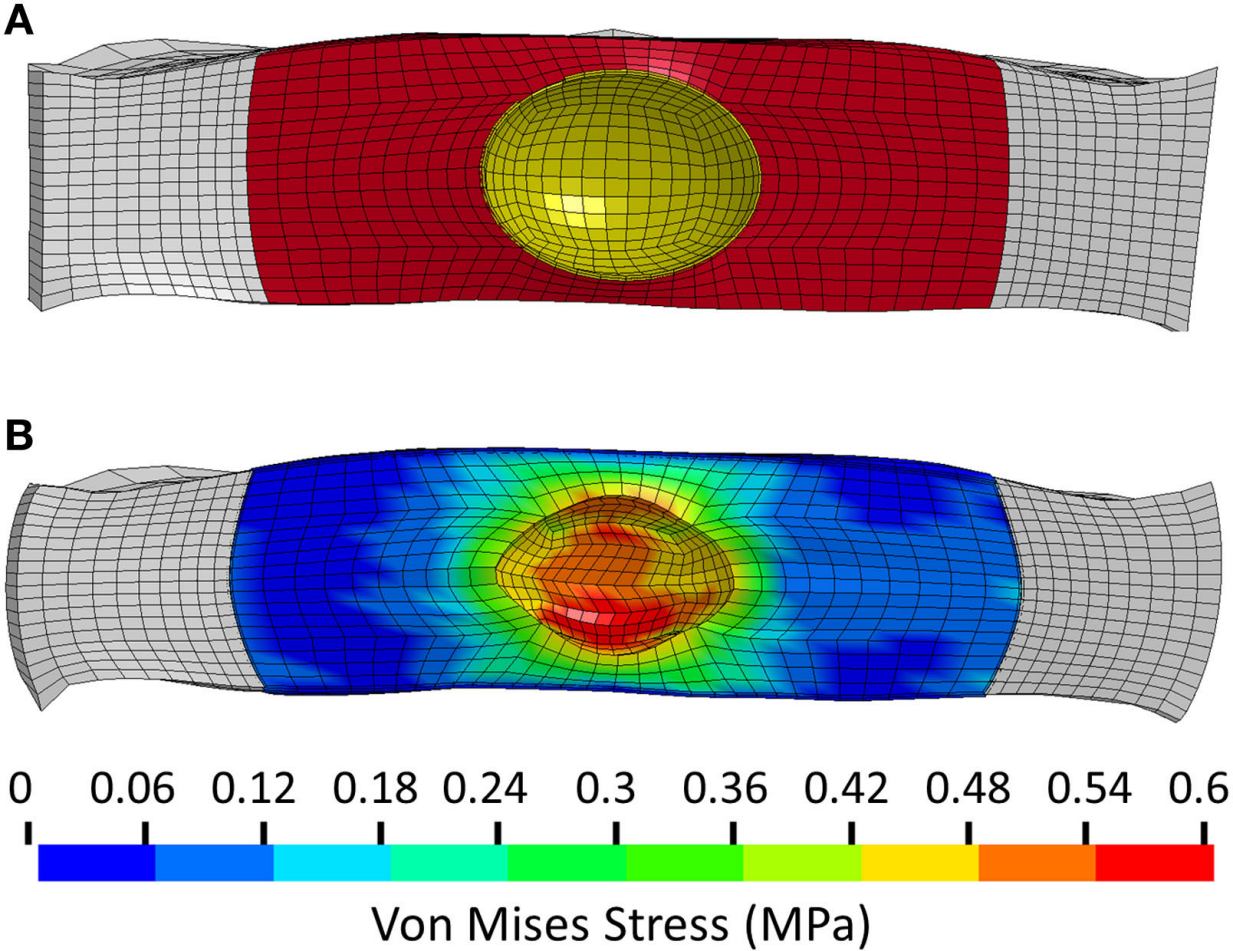
**(A)** Close-up view of the unloaded implant within the annulus. Annulus shown in white, implant outer chamber shown in red, and boundary of inner chamber shown in yellow. **(B)** Stress distribution in the Shore 20A implant following 1200 N axial compression.

The model of the intact normal L3–L4 motion segment had a L3 displacement of 0.9 mm with an axial compression load of 1200 N. The Shore 20A durometer implant with a 0.4 cc inner chamber had slightly higher displacement at the same load (1.1 mm, +22% vs. normal), while the solid implant model had much lower displacement (0.55 mm, −39% vs. normal) (Figure 5). The Shore 30A and 50A durometer implant with chamber had very similar response compared with the Shore 20A durometer implant, while the Shore 75A implant with chamber had displacement between the lower durometer implants and the solid implant (0.74 mm, −17.8% vs. normal). Figure 6 shows a close-up of the Shore 20A implant with chamber, along with the stress distribution within the implant under axial compression, where higher stresses are seen around the inner chamber void. The inner chamber void decreased in size with the application of the compressive load.

**Figure 7:**
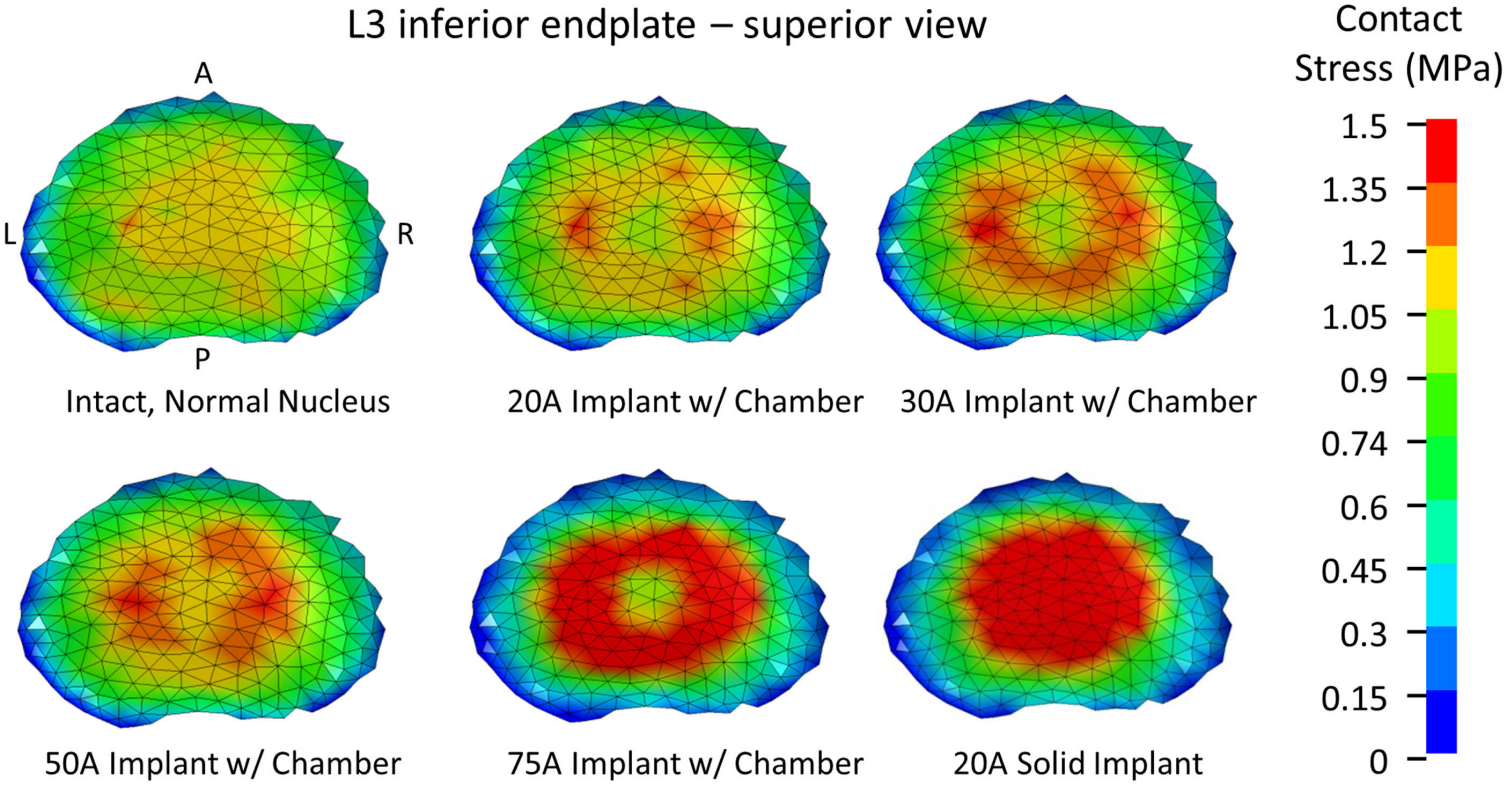
**Endplate stress contours for a 1200 N axial compression**.

For the same axial compressive load, the simulated maximum endplate stress for the intact normal model was 1.3 MPa. The Shore 20A durometer implant with a 0.4 cc inner chamber had slightly lower maximum endplate stress (1.1 MPa, −15% vs. normal) compared to a much higher maximum endplate stress of 2.0 MPa for the solid implant model (+54% vs. normal) (Figure 5). Figure 7 demonstrates that the introduction of the void changes the stress distribution pattern, with less stress in the center of the endplate. The Shore 30A and 50A durometer implant with chamber had very similar response to the Shore 20A durometer implant, with the Shore 75A implant had endplate stress between the lower durometer implants and the solid implant (1.6 MPa, +23% vs. normal) (Figure 5).

**Figure 8:**
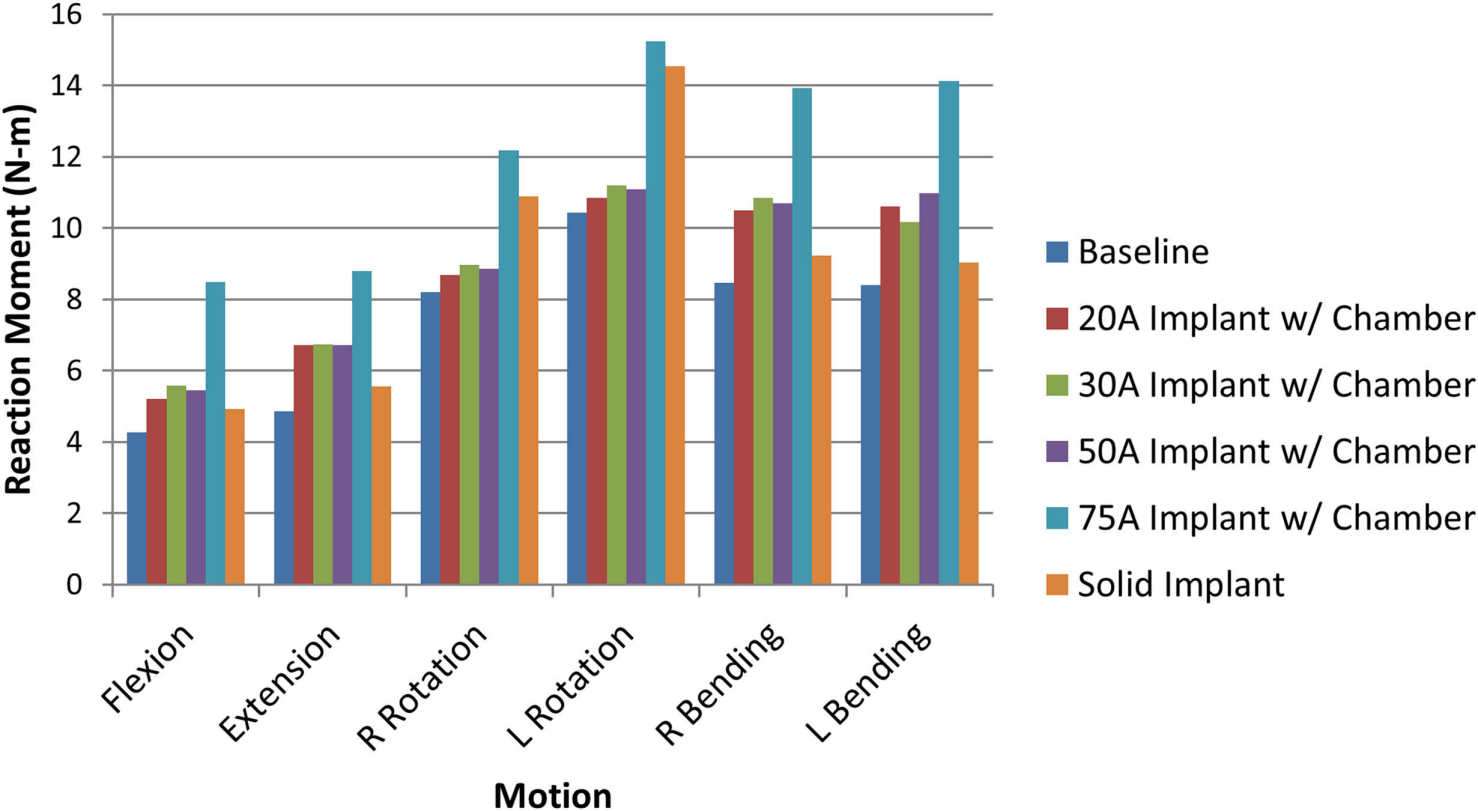
**Resulting reaction moment due to each motion for the intact normal nucleus, implant with chamber, and solid implant**.

**Figure 9:**
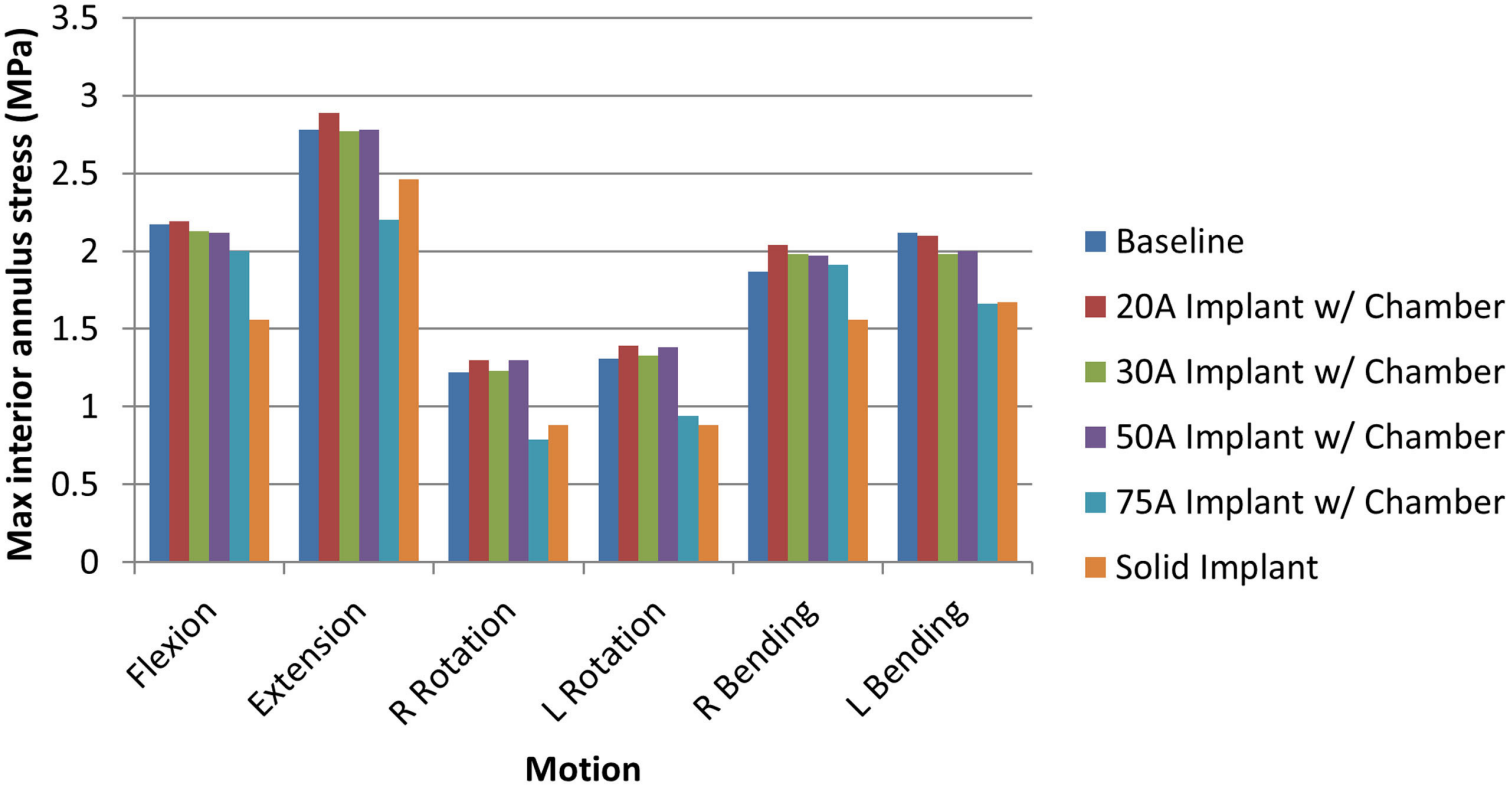
**Maximum interior annulus stress due to each motion for the intact normal nucleus, implant with chamber, and solid implant**.

When comparing reaction moment for the different motions, axial rotation resulted in the largest reaction moment, followed by lateral bending, extension, and flexion for all implant types (Figure 8). Introduction of an implant with chamber resulted in larger reaction moments compared with the intact normal case. The solid implant required a lower reaction moment than the implant with chamber for flexion/extension and lateral bending but a larger moment for axial rotation (Figure 8). The maximum annulus stress on the interior annulus surface was highest for the implant with chamber for all motions except left lateral bending. The solid implant had the lowest annulus stress for all motions (Figure 9). Simulation of the incision through the creation of a slit in the annulus did not have an appreciable effect (<5% difference) on the axial compression, annulus stress, or reaction moment under any loading scenario compared with the model with no incision.

The sensitivity analysis of the silicone durometer showed that, in general, increasing durometer leads to larger reaction moments and lower annulus stress (Figures 8 and 9, respectively). The Shore 20A, 30A, and 50A durometers resulted in similar reaction moments and stresses, while the Shore 75A durometer resulted in larger changes in reaction moment and stress.

## Discussion

An optimally designed nucleus replacement device would result in an IVD that has similar stiffness to that of an intact IVD with a normal nucleus while at the same time creating similar endplate and annulus stresses. An implant that significantly alters the normal physiological response of the motion segment risks causing degenerative effects in adjacent vertebral levels and may also result in subsidence of the device (Herkowitz, 2004).

The model used in this study has been quantitatively validated against experimental data of range of motion due to pure moment loading. Since the experimental data presented here had high variability in axial rotation compared to flexion and lateral bending, the benchmark error metric for axial rotation was much higher. Since the probabilistic error metric is heavily influenced by the experimental variability, the use of a benchmark error metric is important in order to compare the level of validation between different experimental results. For this analysis, the model had the highest probability of having an error less than the benchmark error metric for flexion and the lowest probability for lateral bending. However, an alternate experimental study (Panjabi et al., 1994) had similar response in flexion, extension, and axial rotation, but smaller range of motion in lateral bending than the present study (~4° ± 2°). Using the alternate results would result in a 99 and 100% probability that the error would be less than the benchmark error metric of 1.16 for left and right lateral bending, respectively. The use of small sample sizes in experimental work could mean that the sampled mean and variance is not indicative of the population mean and variance. The simulated response showed lower variance in all rotations, which is likely due to a lack of experimental data on the variance of lumbar disc properties. An assumed CV was used for the disc properties, and a higher CV would lead to a higher variance in the simulated response.

The analysis presented in this study indicates that the Shore 20A, 30A, and 50A durometer silicone nuclear replacement device designed with a 0.4 cc inner chamber void results in a biomechanical response similar to a healthy intact nucleus. Although higher stresses were seen around the inner chamber void under compression, the maximum stress in the device was very low and well below the failure limit of the silicone material. The IVD compression under axial load with these implants was similar to that of the normal disc. Without an inner chamber, the implant was significantly stiffer than a normal nucleus, resulting in a stiffer mechanical response as well as increased stresses on the endplate (Figures 5 and 7). Although the 75A durometer device resulted in similar IVD compression to the normal disc, it also resulted in higher endplate stress, which is not desirable. Nucleus replacement devices that cause endplate stresses significantly higher than those seen in healthy disc are at risk of subsidence (Park and Lakes, 2007). The implant with inner chamber did result in a different stress distribution across the endplate, with lower stresses in the center due to the inner chamber void. However, the range of stresses seen in the implant is similar to that seen with the normal nucleus, so the reduction in stress in the center of the endplate is unlikely to lead to adverse outcomes such as bone resorption.

The analysis also demonstrated that a solid implant reduces stresses on the annulus in all 6 degrees of freedom, which may result in changes to its mechanical properties (Brickley-Parsons and Glimcher, 1984). The device as designed with the inner chamber and 20A durometer silicone resulted in annulus stresses within 10% of the normal disc. However, the solid implant reduced stress in the annulus by as much as 33% in axial rotation. The location of the maximum annulus stress did not change appreciably with the introduction of the implant with chamber or solid implant. The implant resulted in larger reaction moments than the intact normal nucleus, which indicates stability in rotational motions.

As expected, increasing durometer increases the stiffness of the device, leading to increased reaction moments and decreased annulus stresses as the device bears an increasing percentage of the load. Within the range of motion investigated in this study, the Shore 20A, 30A, and 50A durometer silicones exhibited similar biomechanics, showing that their properties were similar in the strain regime studied. The Shore 75A durometer silicone, on the other hand, exhibited larger differences in reaction moment, and stress compared with the intact normal case and the other durometer silicones. This is likely due to the non-linearity of the Shore durometer scale. As seen in Figure 2, the Shore 20A, 30A, and 50A silicones had very similar load profiles, while the 75A silicone had a very different load profile. Physician input was used to decide on the initial configuration utilizing the Shore 20A silicone durometer, and this study did not reveal any biomechanical reasons to change the device durometer.

The results of this study are similar to other studies investigating nucleus replacement devices. Previous work has showed that an optimal nucleus replacement would have a Young’s modulus between 1 and 4 MPa (Rundell et al., 2009; Strange et al., 2010). While the properties of silicone are highly non-linear, in compression, the compressive modulus at 50% strain is ~2.5 MPa.

There are several limitations of this model. Several modeling simplifications were made since reducing model complexity reduces computational time, which is particularly important given the probabilistic nature of the analysis. Ligaments were modeled as non-linear springs, although they would be more accurately represented by continuum elements. The bone was modeled with an isotropic elastic model and the annulus with an isotropic viscoelastic model, which neglects the anisotropic properties of both materials. The isotropic assumption for the disc annulus likely underestimates the true stress in the disc annulus. In addition, the normal model assumes that the nucleus is represented by an elastic fluid, whereas the actual nucleus incorporates poroelasticity. However, the model was quantitatively validated against experimental range of motion data to show the degree of agreement between the model and experiment, with high values of the probabilistic error metric in most loading motions. In addition, the main conclusions of the work are based on a comparative analysis between the normal disc and the disc with the nucleus replacement device. All model simplifications are applied to both models and likely have minimal effect on the comparative differences between models. While the comparison is made to a nucleus that was modeled as an elastic fluid, incorporating poroelasticity may have more of an effect under long-term loading, whereas a relatively short time frame was simulated in this study. In addition, future work will include *in vitro* experimental tests with the implant and further quantitative validation of the computational model will be performed.

The finite element model of the L3–L4 motion segment was able to determine the biomechanics associated with the implantation of a novel nucleus replacement device. The device restores similar biomechanics to the motion segment compared with an intact normal nucleus. In addition, the model was used to perform a sensitivity analysis on device parameters, which relates the device parameters to functional biomechanics. This analysis guides decision making before undertaking expensive and time-consuming experimental animal studies, cadaver studies, or ultimately human clinical trials.

## Author Contributions

JC was responsible for designing, performing, and interpreting the computational study, and drafting and revising the work. WF was responsible for designing and interpreting the computational study, and drafting and revising the work. TE was responsible for performing and interpreting the computational study, and revising the work. TB was responsible for performing the computational study and revising the work. BS was responsible for designing, performing, and interpreting the experimental study, and drafting and revising the work. NY and FP was responsible for designing and interpreting the experimental study, and drafting and revising the work. DN was responsible for designing and interpreting the computational study, and revising the work. All the authors provided final approval of the version to be published and agreed to be accountable for all aspects of the work.

## Conflict of Interest Statement

Southwest Research Institute (authors JC, TE, TB, and DN) received funding from Spinal Stabilization Technologies for this work. Author WF has financial interest in Spinal Stabilization Technologies.
